# A Feline HFpEF Model with Pulmonary Hypertension and Compromised Pulmonary Function

**DOI:** 10.1038/s41598-017-15851-2

**Published:** 2017-11-29

**Authors:** Markus Wallner, Deborah M. Eaton, Remus M. Berretta, Giulia Borghetti, Jichuan Wu, Sandy T. Baker, Eric A. Feldsott, Thomas E. Sharp, Sadia Mohsin, Mark A. Oyama, Dirk von Lewinski, Heiner Post, Marla R. Wolfson, Steven R. Houser

**Affiliations:** 1 Temple University Lewis Katz School of Medicine, Cardiovascular Research Center, Philadelphia, PA United States; 2 Temple University Lewis Katz School of Medicine, Departments of Physiology, Thoracic Medicine and Surgery, Pediatrics, Center for Inflammation, Translational and Clinical Lung Research, CENTRe: Consortium for Environmental and Neonatal Therapeutics Research, Philadelphia, PA United States; 3 School of Veterinary Medicine, University of Pennsylvania, Philadelphia, PA United States; 4 Institute for Translational Medicine and Therapeutics, Perelman School of Medicine, University of Pennsylvania, Philadelphia, PA United States; 5 Division of Cardiology, Department of Internal Medicine, Medical University of Graz, Graz, Austria; 6 Department of Cardiology, Campus Virchow-Klinikum, Charite Universitätsmedizin, Berlin, Germany

## Abstract

Heart Failure with preserved Ejection Fraction (HFpEF) represents a major public health problem. The causative mechanisms are multifactorial and there are no effective treatments for HFpEF, partially attributable to the lack of well-established HFpEF animal models. We established a feline HFpEF model induced by slow-progressive pressure overload. Male domestic short hair cats (n = 20), underwent either sham procedures (n = 8) or aortic constriction (n = 12) with a customized pre-shaped band. Pulmonary function, gas exchange, and invasive hemodynamics were measured at 4-months post-banding. In banded cats, echocardiography at 4-months revealed concentric left ventricular (LV) hypertrophy, left atrial (LA) enlargement and dysfunction, and LV diastolic dysfunction with preserved systolic function, which subsequently led to elevated LV end-diastolic pressures and pulmonary hypertension. Furthermore, LV diastolic dysfunction was associated with increased LV fibrosis, cardiomyocyte hypertrophy, elevated NT-proBNP plasma levels, fluid and protein loss in pulmonary interstitium, impaired lung expansion, and alveolar-capillary membrane thickening. We report for the first time in HFpEF perivascular fluid cuff formation around extra-alveolar vessels with decreased respiratory compliance. Ultimately, these cardiopulmonary abnormalities resulted in impaired oxygenation. Our findings support the idea that this model can be used for testing novel therapeutic strategies to treat the ever growing HFpEF population.

## Introduction

Cardiovascular disease (CVD) is the leading cause of death worldwide, with almost 20% of deaths being cardiac related. By 2030, almost 41% of the US population will be affected by some form of cardiovascular disease^1^. Heart Failure (HF) is the consequence of many forms of CVD and is a major public health problem. Corresponding total direct costs for HF are predicted to increase from $21 billion in 2012 to $53 billion by 2030^2,3^. Novel therapies to reduce the morbidity and mortality of CVD and HF are clearly needed.

HF is classified into three main categories, HF with reduced ejection fraction (HFrEF), HF with mid-range ejection fraction (HFmrEF), or HF with preserved ejection fraction (HFpEF)^4^. HFpEF accounts for about 50% of all cases of HF^3,5,6^ and its prevalence relative to HFrEF is growing by 10% per decade^3^. The underlying pathophysiological initiators as well as factors that drive the progression of HFrEF are well-characterized^7^, in part because there are well-established animal models that adequately mimic the human condition and have reliably predicted the effects of therapeutics that are now known to improve outcomes of HFrEF patients.

Large clinical outcome trials in HFrEF patients have proven the efficacy of neurohumoral inhibition by significantly decreasing mortality^8^. However, the effects of neurohumoral inhibition in HFpEF have consistently failed to reach positive primary outcomes^9–15^. The fact that therapies for HFrEF are not effective in HFpEF patients likely reflects the distinct differences in the pathophysiological mechanisms of the two diseases^16^. What we do know is that the fundamental cardiac pathophysiology of HFpEF is complex and not well established, and truly representative experimental HFpEF models that capture the complexity of this disease have not yet been adequately established^17^. Our view is that the lack of well-established HFpEF animal models has limited the determination of HFpEF therapeutic targets and the preclinical testing of novel therapeutics^18^. There is clearly a need for new HFpEF therapeutics. HFpEF related mortality has not changed over the past 3 decades, with 5-year survival rates as poor as 50%^3,19,20^. While rodent HFpEF models have contributed to our understanding of underlying mechanisms of LV diastolic dysfunction, rodents have inherent limitations due to their size, cardiac structure and function that preclude comprehensive hemodynamic assessments that can be performed in large mammalians and humans. In addition, most of these models develop dilated phenotypes which are more representative of HFrEF than HFpEF. This includes most mouse models of transverse aortic constriction (TAC)^21^. Canine^22,23^ and swine^24,25^ models have revealed important pathophysiological aspects of HFpEF and might be useful, but the majority of preclinical HFpEF models have focused on cardiac function and structure, without characterizing cardiopulmonary interaction, which is a critical component of the HFpEF phenotype.

Dyspnea, particularly during exercise, is one of the cardinal symptoms in HFpEF patients and significantly limits their functional capacity and quality of life. Pulmonary hypertension (PH) is found in 36–83% of HFpEF patients and is strongly associated with morbidity and mortality^26–29^. Furthermore, PH in the early stages of HF has recently become a target of novel therapeutic strategies^30^. Although PH in HF patients is a common and life-threatening complication associated with a poor prognosis, it remains widely underestimated in clinical cardiology^31^. The understanding of PH in HFpEF is still in its early stages. For example, changes in lung parenchyma and lung mechanics that could limit functional capacity and quality of life have not been systematically studied. Many underlying concepts in this field come from observational studies and *in vivo* human data characterizing pulmonary hemodynamics^32,33^. Hoeper *et al*. have suggested that deep phenotyping of animal models with HFpEF cardiopulmonary features will advance the field^34^.

Therefore, the aim of this study was to characterize a feline model of slow-progressive pressure overload to determine if it has critical structural and functional cardiac phenotypic features of HFpEF, and for the first time determine if this model has a HFpEF pulmonary phenotype that includes changes in lung mechanics, gas exchange and hemodynamics. These studies should reveal new mechanistic insights into the development of pulmonary impairment due to elevated left-sided filling pressures and establish this as a suitable model for studying HFpEF mechanisms and testing new therapeutics.

## Results

The aortic banding/sham procedure was performed in 2-month-old male kittens following baseline (BL) echocardiography (ECHO), electrocardiogram (ECG) and collection of blood samples. Animals were studied for 4-months post-banding with serial ECHOs and ECGs performed at 1, 2, 3 and 4-months (see Supplementary Fig. S1). One banded cat was excluded from statistical analyses since the band was shown to be loose with no detectable aortic pressure gradient (measured by invasive FFR method). The body weights (BW) of banded and sham-operated cats did not differ at any time point throughout the entire study (see Supplementary Fig. S1). Some, but not all banded cats, showed signs and symptoms of heart failure (HF) such as tachypnea and diaphragmatic breathing in response to routine physical exertion.

### Echocardiographic Phenotyping

**Figure 1:**
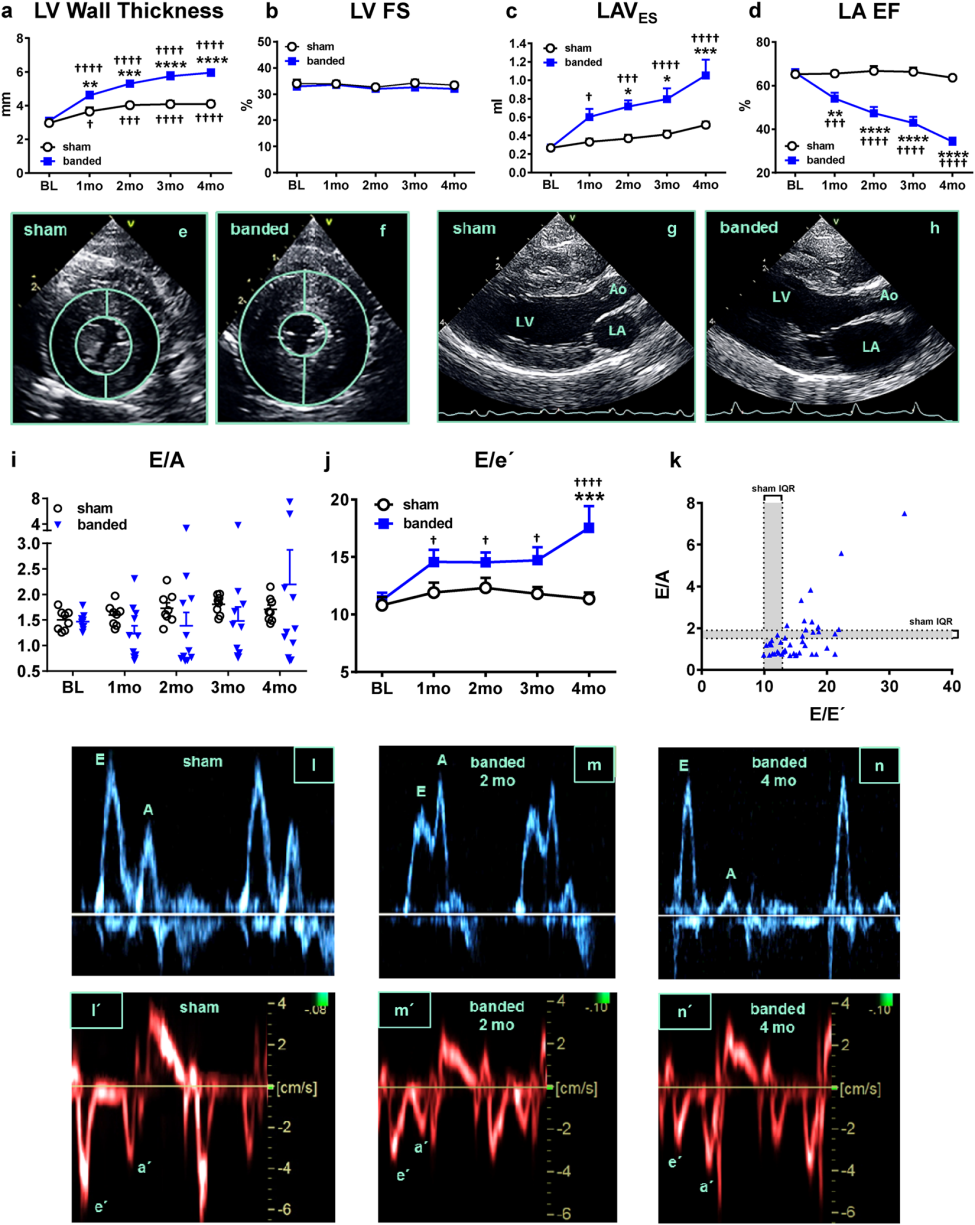
Echocardiographic Phenotyping. Banded cats showed left ventricular hypertrophy **(a)**, preserved global systolic function **(b)**, LA enlargement **(c)** and dysfunction **(d)**, and impaired diastolic function **(i**–**k)**. Representative short **(e–f)** and long **(g–h)** axis b-mode images at 4-months post-banding/sham procedure. E/A ratio **(l**–**n)** and corresponding tissue Doppler-derived peak velocities **(l′**–**n′)**. *p < 0.05, **p < 0.01, ***p < 0.001, ****p < 0.0001 between sham and banded animals. ^†^p < 0.05, ^†††^p < 0.001, ^††††^p < 0.0001 vs BL. Ao = aorta, BL = baseline, E and A = peak early- and late diastolic transmitral velocities, e′ and a′ = spectral tissue Doppler-derived peak early- and late diastolic velocities, FS = fractional shortening, IQR = interquartile range, LA = left atrium, LA EF = left atrial ejection fraction, LAV_ES_ = left atrial end-systolic volume, LV = left ventricle.

Banded cats developed significant concentric left ventricular (LV) hypertrophy, starting at 1-month post-banding and continued to progress throughout the study (Fig. 1a). Global systolic function, assessed by fractional shortening (FS) (Fig. 1b), and left ventricular end-diastolic diameter (LVEDD) (see Supplementary Fig. S2) were unaltered in banded cats. The left atrium showed progressive dilation in banded cats compared to sham cats, as reflected by the increase in left atrial end-systolic volume over time (LAV_ES_: 1.05 ± 0.17 ml vs. 0.52 ± 0.04 ml at 4-months, p < 0.001) (Fig. 1c) and left atrial aortic root ratio (LA/Ao) (see Supplementary Fig. S2). Global LA ejection fraction (EF) was reduced in banded cats at 1, 2, 3, and 4-months compared to sham animals (Fig. 1d). Representative short and long axis b-mode images display LV hypertrophy (Fig. 1f) and LA enlargement (Fig. 1h) at 4-months post-banding, compared to normal cardiac structures in a sham-operated cat (Fig. 1e,g).

LV diastolic function was assessed using pulsed-wave Doppler (PW) (Fig. 1l–n) and tissue Doppler imaging (TDI) (Fig. 1l′–n′) techniques. Banded cats showed different stages of LV diastolic dysfunction (Fig. 1l–n). Some banded cats showed mild diastolic dysfunction (impaired relaxation, grade I) displayed by E/A ratio < 1 (Fig. 1i,m), whereas some presented with a restrictive pattern (high E/A ratio) (Fig. 1i,n), reflective of severe diastolic dysfunction (grade III-IV). Animals with either of these inflow patterns are thought to have diastolic dysfunction. However, some banded cats showed normal E/A ratios, suggestive of normal diastolic function or pseudo-normalization (moderate dysfunction, grade II). Analysis of E/e′, a surrogate for LV end-diastolic pressure (LVEDP)^35^, revealed an increase in banded cats compared to sham cats (Fig. 1j). E/e′ plotted against E/A showed a right- and downward shift in banded cats compared to sham-operated cats (Fig. 1k). All banded cats had abnormal E/A ratios or elevated E/e′ during the study.

### Invasive Hemodynamics

**Figure 2:**
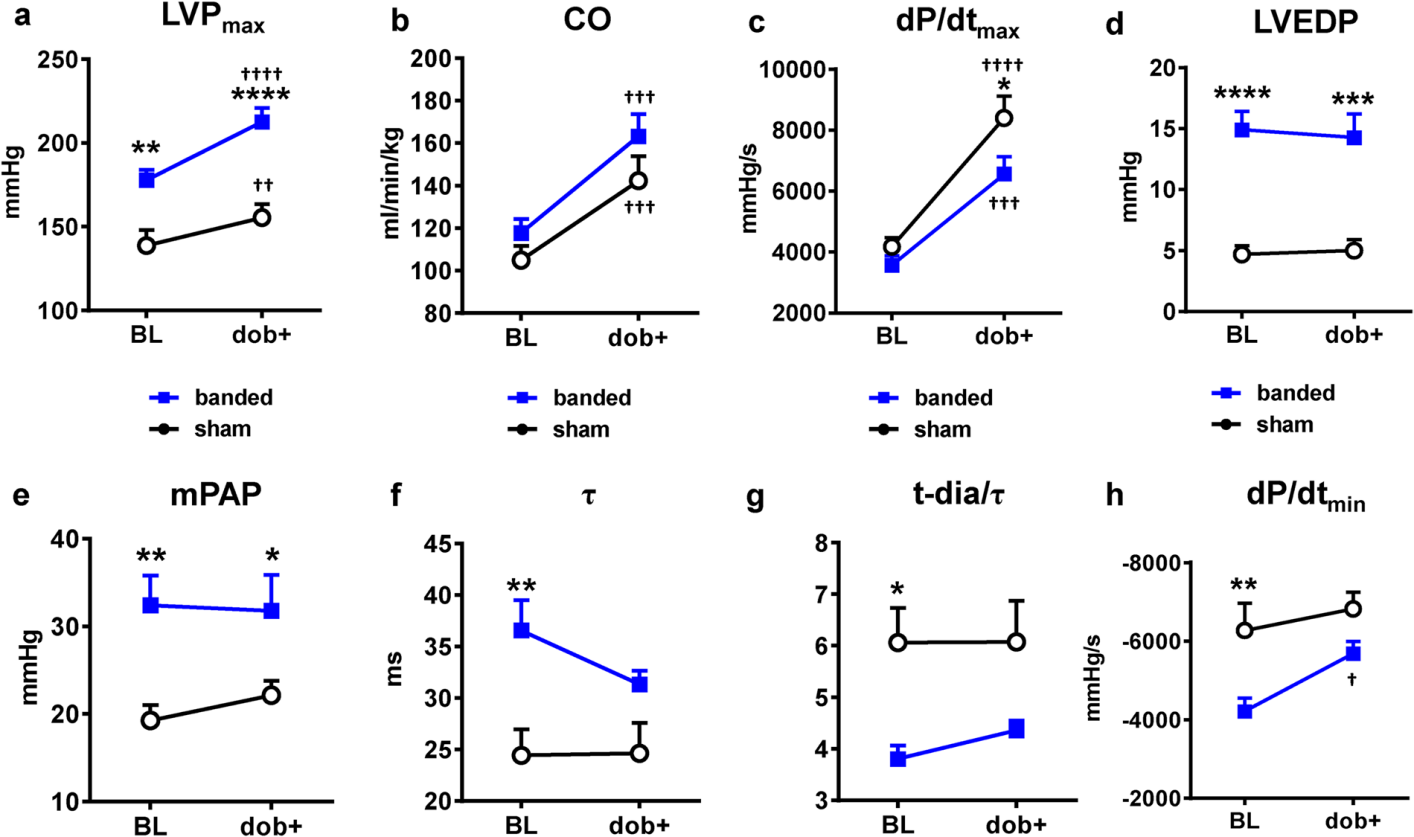
Hemodynamics before and after dobutamine stress testing. **(** **a)** LVP_max_ was higher in banded cats. **(b)** CO was similar between groups. **(c)** Dp/dt_max_ was attenuated in banded animals after dobutamine. Banded animals showed elevated LVEDP **(d)** and mPAP **(e)**. τ was prolonged **(f)**, t-dia/ τ **(g)** and dP/dt_min_ **(h)** were reduced after aortic banding, indicative of impaired relaxation. *p < 0.05, **p < 0.01, ***p < 0.001, ****p < 0.0001 between groups. ^†^p < 0.05, ^††^p < 0.01, ^†††^p < 0.001, ^††††^p < 0.0001 vs BL. BL = baseline, CO = cardiac output, dP/dt_max_ = maximum rate of pressure rise, dP/dt_min_ = maximum rate of pressure decay, LVEDP = left ventricular end-diastolic pressure, LVP_max_ = maximum left ventricular pressure, mPAP = mean arterial pulmonary pressure, τ = isovolumic relaxation constant, t-dia = diastolic time interval.

Terminal hemodynamic and pulmonary function studies were performed at 4-months post-banding. After placing the catheters, baseline measurements including lung mechanics, mixed-venous and arterial blood gas analysis (BGA), cardiac output (CO), pulmonary and cardiac pressures were recorded. Then, functional stress testing was performed by intravenous infusion of dobutamine (5 µg/kg/min). First, we utilized fractional flow reserve (FFR) techniques to measure pressure gradients across the aortic band. The mean systolic aortic pressure gradient was 52 ± 12 mmHg (see Supplementary Fig. S2). The maximum LV pressure (LVP_max_) was significantly higher in banded cats compared to sham cats (Fig. 2a). CO (Fig. 2b) and heart rate (HR) (see Supplementary Fig. S2) increased after dobutamine infusion compared to BL and did not differ between groups. The dP/dt_max_ was comparable between groups at BL, however after dobutamine infusion the increase in dP/dt_max_ was significantly attenuated in banded cats (6548 ± 583 mmHg/s) versus sham cats (8399 ± 714 mmHg/s, p < 0.05) (Fig. 2c). Banded cats had higher left ventricular end-diastolic pressures (LVEDP) (Fig. 2d) and mean pulmonary arterial pressures (mPAP) (Fig. 2e) compared to sham cats at BL and after dobutamine. Dobutamine did not increase LVEDP or mPAP compared to BL in either group. Under resting BL conditions, the LV time constant of isolvolumic relaxation (τ) was prolonged (Fig. 2f) and dP/dt_min_ was reduced (Fig. 2h) in banded versus sham animals at BL, but not after dobutamine. At BL, the ratio between diastolic time interval (t-dia) and LV isovolumic relaxation constant (τ) was also decreased in banded cats (3.8 ± 0.3) compared to sham cats (6.1 ± 0.7; p < 0.05) and slightly increased after dobutamine infusion (Fig. 2g).

### Histological Analyses of LV and Biomarker Levels

**Figure 3:**
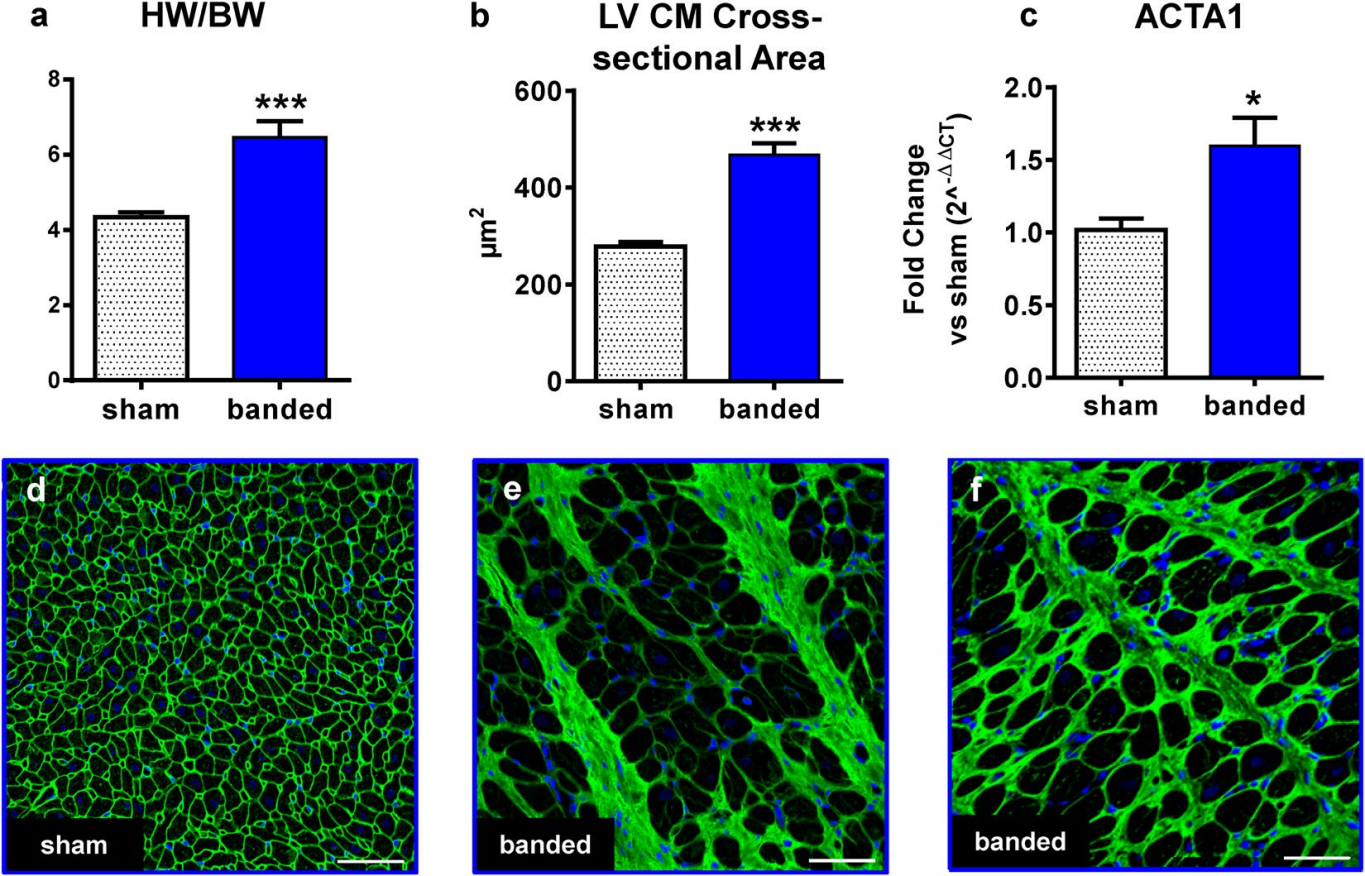
LV hypertrophy. HW/BW ratio **(a)**, CM cross-sectional area **(b)**, and ACTA1 expression levels measured by Real-Time PCR **(c)**, were increased in banded vs sham cats. Confocal micrographs of LV tissue section stained for WGA (green) and DAPI (blue) show increased CM size and extracellular matrix deposition in banded cats **(e–f)** compared to sham **(d)**. Scale bars = 50 µm. *p < 0.05, ***p < 0.001 between groups. ACTA1 = skeletal muscle actin, BW = body weight, CM = cardiomyocyte, HW = heart weight.

**Figure 4:**
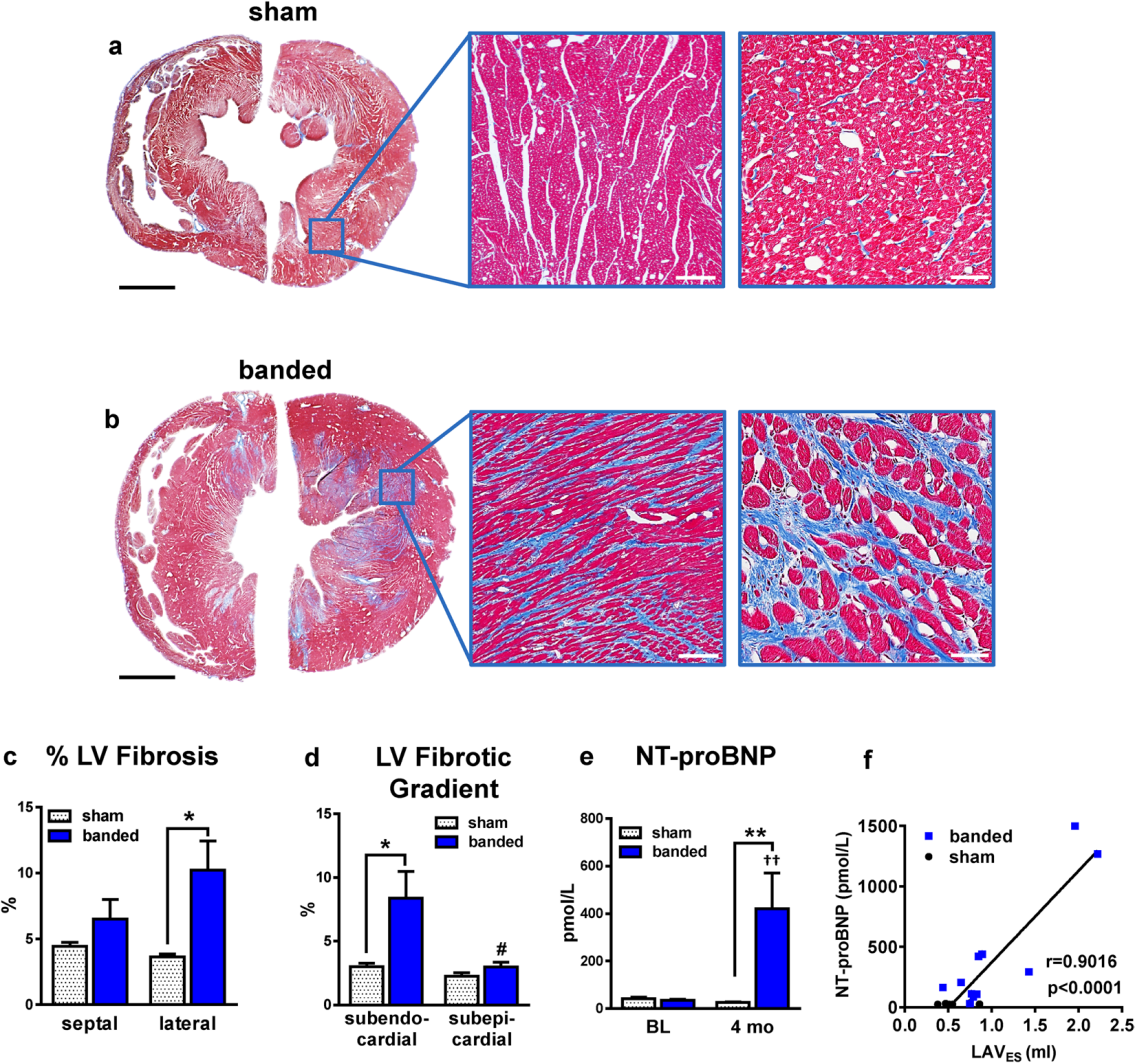
LV fibrosis and NT-proBNP. **(a**,**b)** Bright-field micrographs of LV tissue section stained with Masson’s trichrome indicating an increase in blue-stained fibrotic area in banded **(b)** vs sham **(a)** cats. Scale bars from left to right: 5 mm, 250 µm, and 50 µm. **(c)** Quantification of fibrotic area in septal wall and lateral wall revealed an increase in banded vs sham cats. **(d)** LV fibrosis was more pronounced in subendocardial layers and decreased moving outwards to the subepicardial layers in banded cats. **(** **e** **)** Banded cats had elevated NT-proBNP plasma levels at 4-months post-banding compared to sham cats. **(f)** Correlation between LAV_ES_ and NT-proBNP. *p < 0.05, **p < 0.01 between groups. ^††^p < 0.01 vs BL. #p < 0.05 vs subendocardial. BL = baseline, LAV_ES_ = left atrial end-systolic volume, NT-proBNP = N-terminal pro-brain natriuretic peptide.

The increase in LV wall thickness over time observed by ECHO, reflecting LV hypertrophy, was significantly greater in banded animals and consistent with the results for HW/BW ratio (Fig. 3a), myocyte cross-sectional area (CM CSA) (468 ± 24µm^2^ vs. 278 ± 9µm^2^; p < 0.001) (Fig. 3b), and skeletal muscle actin (ACTA1) expression level (Fig. 3c). Representative confocal micrographs of tissue sections stained with wheat germ agglutinin (WGA) are shown in Fig. 3d–f. LV cross sections were stained for Masson’s Trichrome (MT) and the percentage of aniline blue stained area (collagen) was calculated (Fig. 4c). Lateral and septal walls of the ventricle were analyzed separately and both showed an increase in the percentage of fibrotic area in banded cats compared to sham, but the increase was only statistically significant in the lateral wall (banded: 10.2 ± 2.2% vs sham: 3.6 ± 0.2%, p < 0.01). Total percentage of LV fibrotic area was also increased after aortic banding (banded: 8.4 ± 1.6 vs sham: 4.0 ± 0.3%; p < 0.01). Interestingly, the fibrosis was more pronounced in subendocardial layers compared to subepicardial layers (Fig. D). Figure 4a and b display the differences in fibrotic area between groups and wall segments and furthermore show the fibrotic gradient from subendo- to subepicardium.

Plasma NT-proBNP levels did not differ between groups at BL (banded: 35.4 ± 3.3pmol/L vs. sham: 42.1 ± 3.3pmol/L, p = 0.99). At 4-months banded cats had significantly elevated NT-proBNP levels compared to sham cats (banded: 420.8 ± 179.9 pmol/L = 3566 ± 1270 pg/ml vs. sham: 26.1 ± 1.3 pmol/L = 221 ± 11 pg/ml; p < 0.01) (Fig. 4e). NT-proBNP levels were positively correlated with LA size (LAV_ES_) (r = 0.9, p < 0.0001) (Fig. 4f).

### Functional and Morphological Lung Assessment

**Figure 5:**
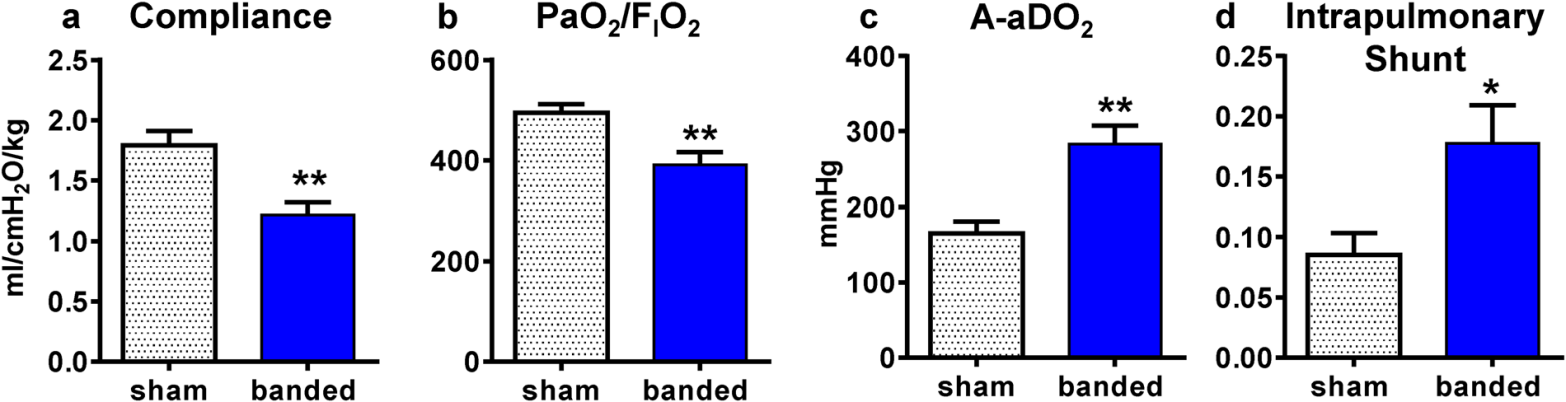
Lung mechanics and gas exchange. **(a)** Respiratory compliance was decreased in banded vs sham cats. **(b**–**d)** Impaired oxygenation in banded vs sham cats was reflected by decreased PaO_2_/F_I_O_2_ ratio, increased A-aDO_2_ and increased intrapulmonary shunt. F_I_O_2_ = 1. *p < 0.05, **p < 0.01 between groups. A-aDO_2_ = alveolar-arterial oxygen difference, PaO_2_ = partial pressure of oxygen in arterial blood.

Pulmonary function was assessed at 4-months post-banding using measurements of pulmonary mechanics and gas exchange. Banded cats had lower respiratory compliance (Fig. 5a) and PaO_2_/F_I_O_2_ ratio (Fig. 5b), and greater alveolar-arterial oxygen difference (A-aDO_2_) (Fig. 5c) and intrapulmonary shunt fraction (Fig. 5d) compared to sham animals.

In these studies, the peak inspiratory pressure (P_insp_) was then adjusted to support a tidal volume of 6–8 mL/kg and target PaCO_2_ within 35–45 mmHg. Banded cats required slightly higher P_insp_ (12.1 ± 0.9 cmH_2_O) compared to sham cats (10.1 ± 0.4 cmH_2_O; p < 0.05) to ensure sufficient ventilation. Under these conditions there was an inverse linear correlation between LVEDP and PaO_2_ (r = −0.63; p = 0.009), as well as between mPAP and PaO_2_ (r = −0.81; p < 0.001) (see Supplementary Fig. S3). Global left atrial function (LA EF) correlated positively with PaO_2_ and respiratory compliance (r = 0.67 and r = 0.72; both p < 0.01) (see Supplementary Fig. S3).

**Figure 6:**
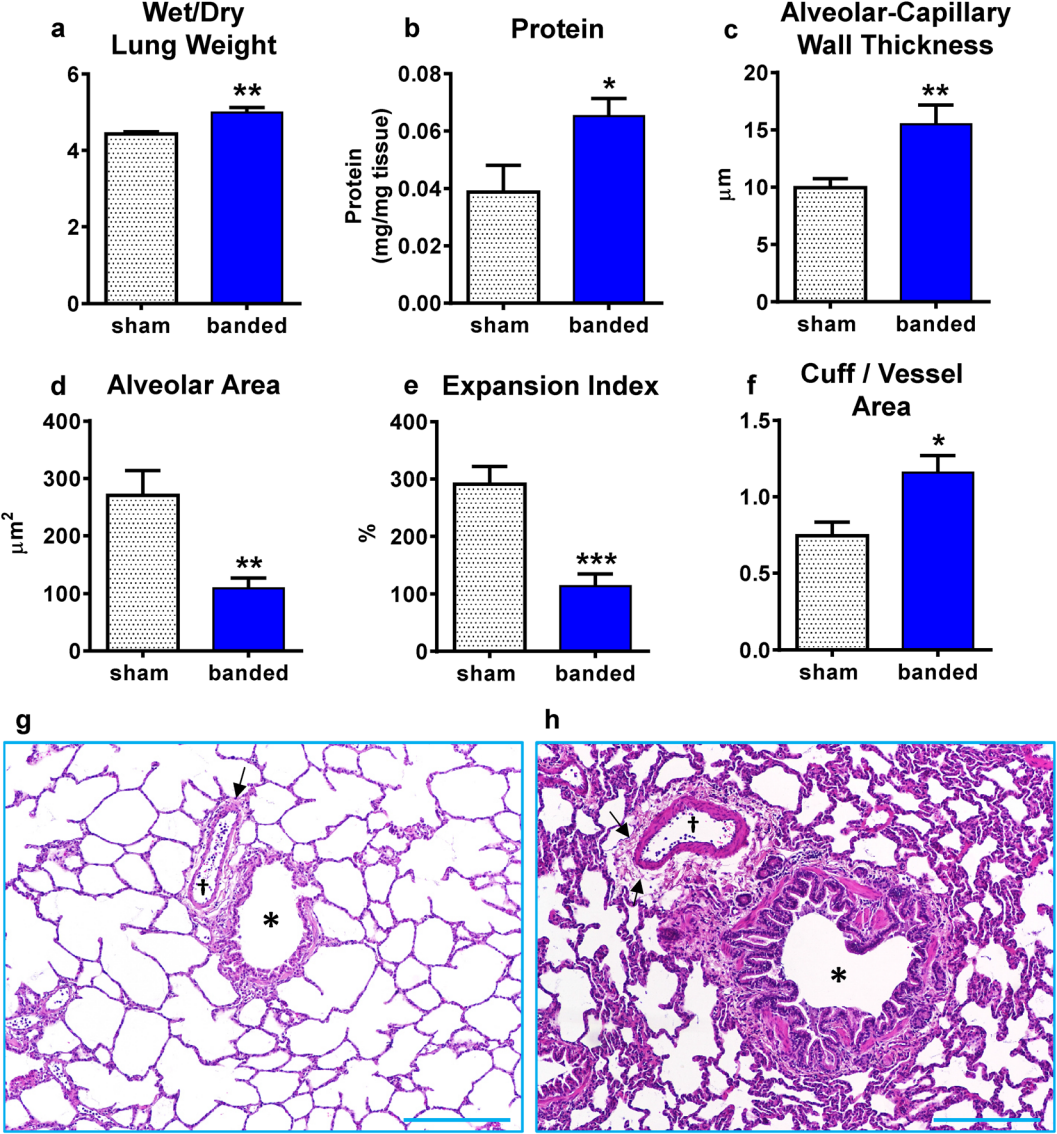
Morphological lung changes. **(** **a**,**b)** Increased wet/dry lung weight ratio and protein content in banded vs sham cats is suggestive of endothelial leaking. **(c)** Alveolar-capillary wall thickness was increased in banded vs sham cats. Alveolar area **(d)** and expansion index **(e)**, the ratio of volume of gas exchange to parenchymal space, were decreased in banded vs sham cats. **(f)** Ratio of cuff/vessel area of extra-alveolar vessels was increased in banded vs sham cats. **(g**,**h)** Bright-field micrographs of H&E-stained lung sections. * = bronchioles, † = extra-alveolar vessel, arrow = fluid cuff around extra-alveolar vessels. Scale bars = 250 µm. *p < 0.05, **p < 0.01, ***p < 0.001 between groups.

Banded cats had an increase in both wet to dry lung weight ratio (Fig. 6a), and protein content normalized to lung weight (Fig. 6b), suggestive of pulmonary edema. Histological analyses of the lung were performed to determine if aortic banding induced morphological changes in the lung that could explain the observed impaired oxygenation, respiratory compliance and increases in lung water. Quantitative histomorphological analysis demonstrated that banded cats had thicker alveolar-capillary walls compared to sham cats (Fig. 6c). The alveolar area (Fig. 6d), alveolar diameter and perimeter (Supplementary Fig. S3) were all smaller in banded cats compared to sham. Furthermore, the expansion index (ratio of volume of gas exchange to parenchymal space) (Fig. 6e) was lower in banded cats compared to sham cats. Assessment of perivascular fluid cuff formation around extra-alveolar blood vessels revealed an increase in banded cats compared to sham cats, independent of vessel size after aortic banding (Fig. 6f, see Supplementary Fig. S3). Representative bright field micrographs of H&E stained sections with histomorphological changes after sham procedure and aortic banding are displayed in Fig. 6g–h.

## Discussion

Our study is the first to perform a comprehensive structural and functional cardiopulmonary assessment in a large animal model of HFpEF. We report novel data that provides mechanistic insights regarding the changes in heart and lung structure and function in this HFpEF animal model. We found that slow-progressive pressure overload in cats resulted in progressive increases in concentric LV hypertrophy, LA enlargement and dysfunction, and LV diastolic dysfunction without LV dilation, which subsequently led to elevated LV filling pressures and pulmonary hypertension with impaired oxygenation and stiffening of the lungs. LV diastolic dysfunction was associated with increased LV fibrosis, cardiomyocyte hypertrophy, elevated NT-proBNP plasma levels, perivascular cuff formation, impaired lung expansion, and alveolar-capillary membrane thickening. According to the European Society of Cardiology (ESC) guidelines, this model meets all criteria necessary for a HFpEF diagnosis^4^ and corresponds to ACCF/AHA stage B/C of HF^36^.

We performed transverse aortic constriction in kittens using a customized pre-shaped band that caused slow-progressive pressure overload as the animals matured. In contrast to most available transverse aortic constriction (TAC) models^37^, which causes an immediate and severe pressure overload, our approach allows the heart to slowly adjust to the increased afterload and does not result in LV dilation and systolic dysfunction. The slow-progressive overload model takes longer to develop but produces a phenotype that more closely mimics the human conditions that can lead to HPpEF. Rodent HFpEF models generally progress to HFrEF over time^21^, which means that HFpEF is just a transient stage in the development of HFrEF. In addition, myocyte properties of rodents, such as regulation of [Ca^2+^] are fundamentally different than in large mammals (cats, dogs, humans, etc.), making translation of results from preclinical HFpEF studies in rodents to humans unreliable^38–43^. In the model developed in the present study, functional (diastolic dysfunction) and structural (LV hypertrophy, LA enlargement) cardiac changes post-aortic banding progressed slowly over time without developing LV dilation.

Echocardiographic and hemodynamic features are the most important components for a HFpEF diagnosis, both of which can be performed in a comprehensive and highly translational fashion in cats. Banded cats developed concentric LV hypertrophy without LV dilation, LA enlargement and dysfunction and LV diastolic impairment, all of which resemble key echocardiographic findings seen in human HFpEF patients^44^. Recently, Zakeri *et al*. reported that diminished LA compliance and reduced contractile function compromise overall cardiac performance in a hypertensive canine model of HFpEF^45^. The same group showed that in human patients with HFpEF, reduced global LA EF and increased LA volume were associated with an increased risk of death^46^. Consistent with these findings, we observed remarkable LA enlargement and reduction in LA EF in banded cats. LA EF was positively correlated with respiratory compliance and PaO_2_. ECHO revealed impaired diastolic function after aortic banding, reflected in abnormal transmitral inflow pattern (E/A ratio) and increased E/e′ ratio, a surrogate for elevated LV filling pressures^47^.

Indices of LV systolic function (FS, dP/dt_max_) were comparable between groups at baseline. Interestingly, after dobutamine infusion the increase in dP/dt_max_ was attenuated in banded cats, supporting characteristics of an impaired systolic reserve. Several studies have reported that patients with HFpEF, despite having preserved ejection fraction, display subtle but significant abnormalities in systolic function at rest when utilizing more sensitive and load-independent techniques^16,48–50^. The subtle impairments in resting systolic function become dramatic during physiological stress^49,51,52^. Limitation in systolic reserve also affects diastolic function, because recoil and suction forces during early diastole are attenuated^53^. Even though dP/dt_max_ varies directly with preload, it is independent of afterload^54^, thus the blunted increase in dP/dt_max_ after dobutamine cannot be explained by a higher afterload in banded cats.

Banded cats showed significant diastolic dysfunction (elevated LVEDP, prolongation of τ, decrease in t-dia/ τ ratio and dP/dt_min_) at rest, which is defined as the inability to fill the LV to an adequate volume at acceptable low pressures^55^. Elevated LV filling pressures are the ultimate downstream expression of LV diastolic dysfunction and provide sufficient evidence to secure a HFpEF diagnosis in symptomatic patients^56^. Additionally, prolongation of τ is a common finding in human HFpEF patients^56–58^. The observed delay in LV isovolumic relaxation in banded cats (prolongation of τ, decrease in t-dia/ τ ratio and dP/dt_min_) will limit LV filling, impair diastolic suction, and increase end-diastolic pressures because relaxation remains incomplete (t-dia/ τ < 3.5)^57,59^. In the present study, T-dia/ τ - ratio in banded cats was 3.8, arguing against incomplete relaxation. However, Schwarzl *et al*. showed in a very elegant study using healthy pigs that incomplete relaxation occurred at ratios clearly higher than 3.5^60^. Isovolumic active relaxation is related to myofilament dissociation and ATP-dependent calcium reuptake^53^ and is a sensitive marker for myocardial ischemia^61^. Therefore, coronary microvascular dysfunction could have caused prolongation of τ in banded cats with significant LV hypertrophy.

Exercise intolerance is one of the hallmark clinical features of HFpEF and is attributed to impairments in systolic and diastolic reserve, chronotropic incompetence and peripheral autonomic and vascular dysfunction^62^. Although, full exercise stress testing was beyond the scope of the current study, some insight about native activity can be appreciated from observation of the unrestrained cats who were group housed in rooms allowing free movement and social interaction. In this regard, banded cats presented signs and symptoms of HF such as abnormal breathing patterns (abdominal efforts, diaphragmatic breathing) and higher respiratory rates, particularly after physical exertion. As an alternative to controlled exercise, we used pharmacologic stress testing. Dobutamine infusion did not cause the hemodynamic profiles in banded cats to decline. LVEDP and mPAP did not change and active relaxation improved in response to dobutamine, which is not surprising since ß-adrenoreceptor signaling accelerates relaxation and therefore counteracts acquired diastolic dysfunction^63^. Our findings are also consistent with the results of a hemodynamic study performed in human HFpEF patients. Penicka *et al*. reported a decrease in both LVEDP and τ, and an increase in dP/dt_min_ after dobutamine infusion, while performing hand-grip exercises increased LVEDP and caused a prolongation of τ^64^.

HFpEF is associated with interstitial fibrosis, which is mostly reactive^65^ and a result of fibroblast activity and extracellular matrix remodeling^66^. We consistently found that both lateral and septal LV walls of the banded group had a higher percentage of fibrosis compared to the sham group. Interestingly, the fibrosis was diffuse, and more pronounced in the lateral free wall compared to septal wall in banded cats. This difference in fibrosis between the LV walls in HFpEF has not to our knowledge been reported elsewhere. Furthermore, the LV fibrosis was more pronounced in the subendocardium vs the subepicardium. A similar pattern was observed in dogs with LV hypertrophy and failure induced by chronic pressure-overload and related to a limited subendocardial coronary reserve^67^ and latent ischemia during adrenergic stimulation^68^. In line with that, isovolumic relaxation is prolonged in banded cats, which could relate to less potential recoil energy stored in the damaged matrix of the subendocardium during systole^69^. It is likely that latent myocardial ischemia during pressure-overload is a major trigger for primarily subendocardial fibrosis also in our HFPEF model. We speculate that LV fibrosis in HFpEF might evolve with a subendo- to subepicardial gradient, similar to the wavefront of myocardial infarction during acute coronary occlusion^70^. NT-proBNP, a surrogate marker for increases in atrial and ventricular size as well as cardiomyocyte stretch, has been validated in cats and is released in proportion to the degree of stretch and stress on the myocardium^71^. The ESC defined elevated natriuretic peptides (BNP, NT-proBNP) as a criterion to secure a HFpEF diagnosis^4^. Due to the high LV filling pressures and significant LA enlargement in banded cats, it was not surprising that we found elevated NT-proBNP levels at 4-months post-banding, which further correlated positively with LA size. Oyama *et al*. reported that in cats with respiratory symptoms, plasma NT-proBNP levels > 270 pmol/L are suggestive of HF as the likely cause with high sensitivity and specificity^71^. Hypertensive heart disease (HDD) without heart failure and HFpEF share common features, such as LVH and diastolic dysfunction, but there are also distinct differences between these two entities. Zile et al. reported that HFpEF patients had increased LVEDP, LA volume, NT-proBNP levels, and myocardial passive stiffness compared to both control and HDD without HF patients. In comparison to control patients, HDD without HF patients had no change in myocardial passive stiffness or LVEDP. The authors also showed that NT-proBNP levels were elevated in HDD compared to controls and these levels were further increased in HFpEF patients^72^. A recent study^73^ in hypertensive cats reported NT-proBNP levels of 236 pmol/L, which were measured by the same commercial laboratory that we used. Our HFpEF cats had higher NT-proBNP levels, which is consistent with the findings reported by Zile *et al*.^72^. We did not perform passive stiffness experiments, but all our banded cats had increased LV filling pressures. Speckle-tracking based longitudinal strain has been shown to discriminate between HFpEF and hypertensive patients with diastolic dysfunction but no HF. In comparison with hypertensive patients, HFpEF patients had significantly lower longitudinal strain values^74^. Unfortunately, STE analyses in cats are not well established and poorly validated.

**Figure 7:**
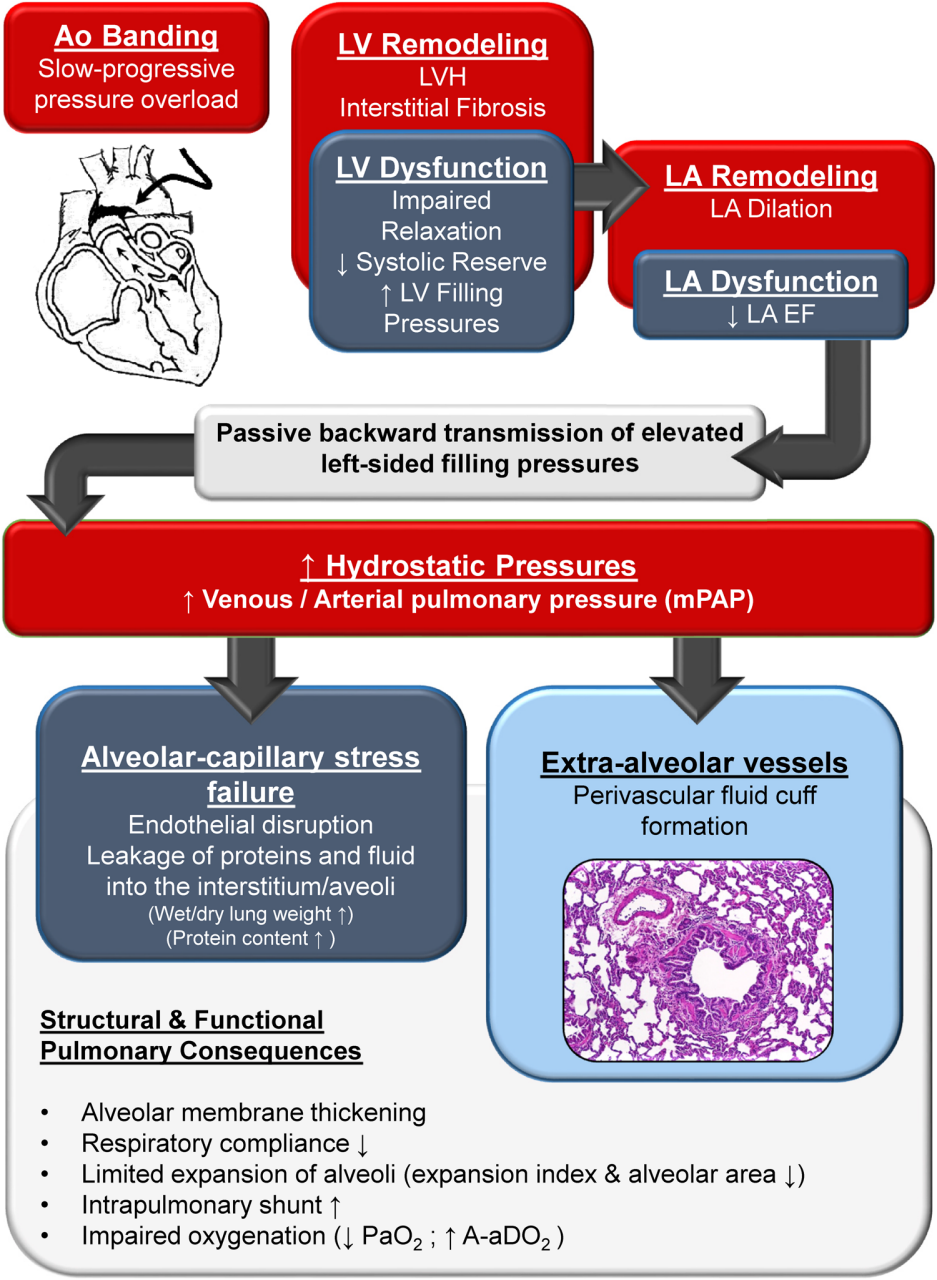
Concept of pathobiological and functional abnormalities due to elevated left-sided filling pressures. Slow-progressive pressure overload induces LV hypertrophy and interstitial fibrosis which subsequently causes impaired relaxation (prolongation of τ, decreased dP/dt_min_) and impairment in systolic reserve function. LV remodeling and dysfunction results in elevated LV filling pressures (LVEDP), LA enlargement and LA dysfunction. Passive backward transmission of elevated pressures causes an increase in hydrostatic venous pressure and leads to pulmonary arterial hypertension, which promotes endothelial disruption with protein and fluid loss into the interstitium and alveoli (alveolar-capillary stress failure). Interestingly, not only capillaries are affected, but extra-alveolar vessels also show distinct perivascular fluid cuff formation. These cardiopulmonary abnormalities result in decreased respiratory compliance and impaired oxygenation. A-aDO_2_ = alveolar-arterial oxygen difference, Ao banding = aortic banding, LA = left atrium, LVH = left ventricular hypertrophy, mPAP = mean pulmonary arterial pressure, PaO_2_ = partial pressure of oxygen in arterial blood. Red boxes = structural changes, dark blue = functional changes, light blue = novel morphological pulmonary finding.

Largely due to limitations of current available HFpEF animal models, current knowledge of pulmonary hypertension (PH) in HFpEF is derived from *in vivo* human pulmonary hemodynamic studies. Furthermore, histological data supporting vascular remodeling in HFpEF is lacking^33^. In this study, we present a pre-clinical model of HFpEF with PH that allowed us to assess the structural and functional pulmonary changes as a result of high left-sided filling pressures and provides new mechanistic insights about PH in HFpEF. Our concept of pathobiological and functional abnormalities is displayed in Fig. 7. Slow-progressive pressure overload in cats led to LVH, LV fibrosis and LV diastolic impairment, which caused elevated LVEDP and LA enlargement. Backwards transmission of elevated left-sided filling pressures caused an increase in hydrostatic venous pulmonary pressure and pulmonary arterial hypertension (increased mPAP), ostensibly due to approaching the elastic limits of the compliant pulmonary vasculature. According to the most recent PH guidelines, the combination of elevated LVEDP ( > 15 mmHg) and mPAP ( ≥ 25 mmHg) is classified as post-capillary PH, which is further subdivided into isolated post-capillary PH (Ipc-PH) and combined post- and pre-capillary PH (Cpc-PH)^30^. The observed increase in wet/dry lung weight ratio and the elevated protein content following aortic banding are suggestive of capillary endothelial disruption and impaired permeability due to increased hydrostatic pressure with subsequent interstitial edema and protein loss from the vasculature into the lung tissue proper. These reversible changes have been described in Ipc-PH and are summarized under the term ‘alveolar-capillary stress failure’^31^. In addition, we found extra-alveolar vessel abnormalities including perivascular fluid cuff formation, impaired expansion of the lung (decreased expansion index), smaller alveolar areas, and thickening of alveolar-capillary membrane in banded cats. To the best of our knowledge, this is the first time perivascular cuff formation has been described in HFpEF PH. Lowe *et al*. reported perivascular fluid cuff formation along extra-alveolar vessels without alveolar flooding or blood gas abnormalities in a lung injury model with thapsigargin. The authors showed that perivascular fluid cuffs reduced lung compliance by increasing tissue resistance and impairing mechanical coupling between bronchovascular bundle and the lung parenchyma^75^. Consistent with these findings, we also observed cuff formation and stiffening of the lungs. Finally, these changes led to an increase in intrapulmonary shunt fraction and impaired oxygenation.

## Conclusion

The present experiments showed that slow-progressive pressure overload of the LV caused LV concentric hypertrophy, diastolic dysfunction with elevated LV filling pressures, and LA dilation with reduced LA systolic function. These are critical features of HFpEF. In addition, our experiments document that the HFpEF phenotype included early stages of PH (Ipc-PH) with endothelial stress failure affecting not only the alveolar-capillary membrane but also extra-alveolar vessels, which subsequently causes significant pulmonary functional impairments with impaired oxygenation and respiratory symptoms. Since most of the pulmonary changes may likely be reversible at this stage, effective therapeutics could yield very promising outcomes. As highlighted by Hoeper *et al*. there is an imbalance between the urgent need to treat PH-HFpEF safely and effectively and a lack of conclusive scientific evidence supporting its presence. Collectively, our findings support the translational efficacy of this animal model and support its use as a platform to test novel therapeutic strategies to treat the ever growing HFpEF population.

### Limitations

We did not collect data on pressure volume (PV) loops due to technical difficulties. PV catheters that are used for animals this size are 3 F and bigger. A 3 F catheter would very likely compromise aortic blood flow through the band and make comparison between sham and banded cats difficult. Therefore, we used a 2 F micro-tip pressure catheter (SPR-320, Millar) which is used for hemodynamic studies in rodents in order to minimize the effects on the hemodynamic study. RV function is known to be abnormal in HFpEF, but was not assessed is this study. Echo based imaging parameters, such as TAPSE, MAPSE, and speckle-tracking based strain analyses are used to assess RV and LV function, but are not well established and validated in cats. Invasive hemodynamics were performed in anesthetized cats in the supine position, which is an unusual posture for quadrupeds. Dobutamine stress testing does not perfectly replicate physical exercise, therefore results need to be interpreted with caution. Furthermore, it is well established that with normal aging LV stiffness increases, diastolic relaxation becomes compromised and diastolic suction is impaired, which may further compromise exercise tolerance in human HFpEF patients^16^. However, the cats in our study are still young (age 6 months) at the time they undergo hemodynamic testing, and except for hypertension, do not share other comorbidities and risk factors of human HFpEF patients. It is likely that in contrast to young cats, aged cats would have impaired cardiovascular reserve. Another limitation of the study is that once the aortic constriction surgery is performed, the band is in place for the entire duration of the study since it cannot easily be removed. Furthermore, the pericardial sack is dissected during both the sham and aortic constriction procedures, which could have potentially resulted in an underestimation of the left-sided filling pressures^76^.

## Methods

### Please refer to the Supplementary Information (online) for a detailed description of experimental methods

#### Banding Procedure

All animal procedures were approved by the Temple University Lewis Katz School of Medicine Institutional Animal Care and Use Committee and all experiments were performed in accordance with relevant guidelines and regulations. We utilized a total of 20 male short hair kittens, aged 2 months (1.3 kg), that underwent either aortic constriction (n = 12), with customized pre-shaped bands, or a sham procedure (n = 8). The pre-shaped band was placed around the ascending aorta and gently tied down without causing significant constriction of the aorta.

#### Transthoracic Echocardiography (ECHO)

Echocardiography (ECHO) was performed with a Vivid q Vet Premium BT'12 using a 12S-RS sector probe at baseline and 1, 2, 3, and 4-months post-surgery. Echocardiographic measurements were subsequently performed offline in a blinded fashion with EchoPAC SW v201.

#### Hemodynamic Studies

At 4-months post-surgery, comprehensive hemodynamic studies were performed. In order to perform cardiopulmonary measurements under comparable intrathoracic pressure profiles and free of spontaneous respiratory efforts, anesthesia and paralysis were maintained with sodium pentobarbital (10 mg/kg/hr) and pancuronium bromide (0.1 mg/kg/hr), respectively. Animals were initially supported (Dräger Babylog 3000) with the same settings (Pinsp = 10 cmH_2_O, PEEP = 3 cmH_2_O, respiratory rate = 16 breaths/min, F_I_O_2_ = 100%, I:E = 1/4.5) of time-cycled, volume-controlled, pressure-limited ventilation, independent of study group, and data generated from the integrated pulmonary mechanics module utilizing airway manometry and pneumotachography were recorded. The peak inspiratory pressure was then adjusted to support a tidal volume of 6–8 mL/kg. Arterial blood samples were analyzed and respiratory phase timing was adjusted to maintain PaCO_2_ within 35–45 mmHG, with all other ventilator settings remaining constant, and the animals were then fully instrumented (see supplemental). Trans-aortic band pressure gradients were measured invasively using the Fractional Flow Reserve (FFR) module (RadiAnalyzer Xpress, St. Jude Medical Inc., MN, USA). Pulmonary pressures were measured through the lumen of a thermodilution catheter (5 Fr). LV pressure was measured with a 2 F micro-tip pressure catheter (SPR-320, Millar Instruments, Houston, TX) and cardiac output (CO) was determined using thermodilution methodology (9520A-American Edwards Laboratories, Inc.) Measurements were recorded at end-expiration with a steady PEEP of 3cmH_2_O. Afterwards, dobutamine was infused at 5 µg/kg/min and all measurements outlined above were performed again. Data were acquired and analyzed using Powerlab and LabChart Pro 8.1.5 (ADInstruments, CO, USA). The datasets generated during and/or analysed during the current study are available from the corresponding author on reasonable request.

#### Statistical Analysis

Data management and statistical analyses were performed using Graph Pad PRISM 6.07. Values are expressed as mean ± SEM. Dependent variables were considered to be continuous. A mixed - effects model was used throughout. ECHO and hemodynamic variables were analyzed based on an analysis of variance multifactorial design (time: BL, 1, 2, 3, 4-months post-surgery; treatment: sham vs banded) with repeated measures on time. Treatment group means were compared vertically using the Dunn-Bonferroni procedure and horizontally with their respective baseline. Group comparisons for all other parameters were performed using the Mann-Whitney test. Statistical significance was accepted at p < 0.05.

#### Data availability

The data that support the images and plots within this paper and other findings of this study are available from the corresponding author upon reasonable request.

## Electronic supplementary material

Supplementary Information
