# Supplementary material for: A Feline HFpEF Model with Pulmonary Hypertension and Compromised Pulmonary Function
Source: Sci Rep. 2017 Nov 29;7:16587. doi: 10.1038/s41598-017-15851-2 (PMC5707379; doi:10.1038/s41598-017-15851-2)
Supplement: Supplementary file 1 — Supplementary Information [file 41598_2017_15851_MOESM1_ESM.pdf]

A Feline HFpEF Model with Pulmonary Hypertension and Compromised Pulmonary Function

Markus Wallner[1,5], MD, PhD; Deborah M. Eaton[1], BS; Remus M. Berretta[1], BS; Giulia Borghetti[1], PhD; Jichuan Wu[2], MD, PhD; Sandy T. Baker[2], MLAS; Eric A. Feldsott[1], BS; Thomas E. Sharp III[1], PhD; Sadia Mohsin[1], PhD; Mark A. Oyama[3,4], DVM; Dirk von Lewinski[5], MD; Heiner Post[6], MD; Marla R. Wolfson[2], PhD; Steven R. Houser[1], PhD

[1]Temple University Lewis Katz School of Medicine, Cardiovascular Research Center, Philadelphia, PA; [2]Temple University Lewis Katz School of Medicine, Departments of Physiology, Thoracic Medicine and Surgery, Pediatrics, Center for Inflammation, Translational and Clinical Lung Research, *CENTRe*: Consortium for Environmental and Neonatal Therapeutics Research, Philadelphia, PA; [3]School of Veterinary Medicine, University of Pennsylvania, Philadelphia PA; [4]Institute for Translational Medicine and Therapeutics, Perelman School of Medicine, University of Pennsylvania, Philadelphia PA; [5]Division of Cardiology, Department of Internal Medicine, Medical University of Graz, Graz, Austria; [6]Department of Cardiology, Campus Virchow-Klinikum, Charite Universitätsmedizin, Berlin, Germany

Corresponding Author:

Steven R. Houser, PhD
Lewis Katz School of Medicine, Temple University
Cardiovascular Research Center
3500 N. Broad St. MERB 1080C
Philadelphia, PA 19140
Phone: 215-707-3278
Fax: 215-707-0170
Email: srhouser@temple.edu

Supplementary Information

Detailed Methods

Animal Procedures: All animal procedures were approved by the Temple University School of Medicine Institutional Animal Care and Use Committee. We utilized a total of 20 male short hair kittens (aged 2 months (1.3kg)) (Liberty Research Inc., Waverly, NY), that underwent either aortic constriction (n=12), with customized pre-shaped bands or a sham procedure (n=8). Animals were sedated with ketamine (25mg/kg) and acepromazine (0.1mg/kg), intubated, and mechanically ventilated (Narkomed 2b). Surgical (plane) anesthesia was maintained throughout the procedure with 1-2% isoflurane mixed with 100% oxygen. Under sterile conditions, a 2-4 cm skin incision was made over the 3rd-4th intercostal space and extended through the intercostal muscles. The pericardium was opened and the aorta was dissected from the pulmonary artery. The pre-shaped band was placed around the ascending aorta and gently tied down without causing significant constriction of the aorta. Animals received heat support during anesthesia and recovery. In contrast to immediate pressure overload, this approach results in a slow progressive pressure overload during growth. Cats were group housed in spacious rooms that were enriched with scratching posts, climbing towers, and hammock where they could freely move and were provided with food and water ad libitum.

Transthoracic Echocardiography (ECHO): Echo was performed with a Vivid q Vet Premium BT'12 using a 12S-RS sector probe at baseline and 1, 2, 3, and 4 months post-surgery. Animals are sedated with an i.m. injection of alfaxalone (1mg/kg), butorphanol (0.5mg/kg) and midazolam (0.5mg/kg). Alfaxalone is a neuroactive steroid molecule with properties of a general anesthetic. Alfaxalone does not cause an increase in HR, which is occasionally observed with ketamine. Ivabradine (0.3mg/kg) was administered intravenously prior to echo to decrease the heart rate to around 150 bpm to ensure reliable diastolic assessment (1). Echocardiographic measurements were subsequently performed offline in a blinded fashion with EchoPAC SW v201. Left ventricular (LV) wall thickness was measured at end-diastole by calculating the mean of LV anterior ventricular septum and posterior wall diameter. The left atrial aortic root ratio (LA/Ao) was measured in early ventricular diastole using the first frame after aortic ejection using the right parasternal short axis view (2). LA volume (LAV) and LA area were calculated as described in humans (3) from a right parasternal long-axis view, using the Simpson's rule at end-systole and end-diastole. LA function, assessed by LA volumes, was calculated according to atrial function studies in humans (4). Changes in LAV expressed as ejection fraction (LA-EF = (LAVmax - LAVmin)/LAVmax) (5). LV end-diastolic diameter and fractional shortening (FS) were measured in b-mode from a right parasternal short-axis view. LV outflow tract (LVOT) diameter was measured in b-mode from a parasternal long-axis view in early systole. The LVOT velocity time integral was measured from an apical 5 chamber view. Stroke volume (SV) was calculated as SV= Pi(LVOT diameter/2)^2 x LVOT VTI and cardiac output (CO) was calculated as CO=HRxSV. Diastolic function was determined using pulsed-wave Doppler (PW) and tissue Doppler imaging (TDI) techniques. Using an apical long-axis view, transmitral inflow velocities were recorded by setting the sample volume in the mitral orifice close to the tip of the mitral leaflets. Spectral waveforms were analyzed for peak early- and late-diastolic transmitral velocities (E and A waves) and E/A was calculated. Mitral inflow data from cats with constant, complete EA wave summation were excluded from further analysis. When partial EA fusion was present, data was included provided that the A wave commenced during the E deceleration at less than 0.2 m/s (E at A). Recordings of PW-TDI of the basal interventricular septum (IVS) and left ventricular free wall (LVFW) were used for measurements of the major TDI velocities during ventricular diastole (E´) (6). The average of septal and lateral E´ was calculated and used for E/E´.

Hemodynamic Studies and Pulmonary Function Assessment: 4 months post-surgery, comprehensive hemodynamic studies were performed. Sodium pentobarbital was administered intravenously (20mg/kg) for induction and the plane of anesthesia was maintained with 10mg/kg/hr sodium pentobarbital. In order to perform cardiopulmonary measurements under comparable intrathoracic pressure profiles between animals and eliminate spontaneous respiratory efforts, the cats were paralyzed with 0.1 mg/kg/h pancuronium bromide, orally intubated with a cuffed endotracheal tube (3.0mm, Medline,

Industries, IL, USA), and supported with time-cycled, volume-controlled, pressure-limited ventilation (Babylog 8000 plus, Dräger Lubeck, Germany) using the following initial settings: F_IO_2=1, peak inspiratory pressure = 10 cmH_2O, positive end expiratory pressure = 3 cmH_2O, inspiratory flow = 4 L/min, inspiratory time = 0.66 sec, expiratory time=3 sec. Baseline arterial blood samples were analyzed, and data generated from the integrated pulmonary mechanics module utilizing airway manometry and pneumotachography as fitted to the equation of motion were recorded. Then peak inspiratory pressure was adjusted to support a tidal volume of 6-8 mL/kg. Arterial blood samples were reanalyzed and respiratory phase timing was adjusted to target $PaCO_2$ within 35–45 mmHG, with all other ventilator settings remaining constant. A balanced crystalloid infusion (Ringer's-Lactate) was administered at a fixed rate of 10ml/kg/hr intravenously throughout the study. A body temperature of 38–39°C was maintained using a warming pad. ECG, SPO_2 and temperature were continuously monitored. Sheaths were placed in the right femoral vein and artery, left carotid artery and external jugular. Animals were instrumented with a 5F pulmonary artery flotation catheter (Arrow thermodilution catheter, AI-07165, Irvine, CA), a 2F LV pressure catheter (SPR-320 Mikro-Tip), and a 3F pig tail catheter was placed in the descending aorta. All catheter placements were performed under fluoroscopic guidance. Data was acquired using Powerlab and LabChart Pro 8.1.5 (ADInstruments, CO, USA). Hemodynamic data was analyzed offline using LabChart Pro 8.1.5 (ADInstruments, CO, USA)

Experimental Protocol: All animals were allowed to stabilize for at least 30 minutes following instrumentation. Trans-aortic band pressure gradients were measured invasively using Fractional Flow Reserve (FFR) module (RadiAnalyzer Xpress, St. Jude Medical Inc., MN, USA), which is used in humans for coronary physiology. Pulmonary pressures were measured with physiological pressure transducers (MLT844, ADInstruments, CO, USA). Cardiac output (CO) was determined by thermodilution methodology (9520A-American Edwards Laboratories, Inc.). Steady-state hemodynamics were acquired at spontaneous heart rate. These measurements were recorded at end-expiration with a steady PEEP of 3cmH_2O to minimize respiration-induced changes of intrathoracic pressure. Afterward, dobutamine was infused at 5µg/kg/min. All measurements outlined above were repeated, and then dobutamine infusion was stopped.

Heart Tissue Collection and Processing: A cardiectomy was performed, then the heart was rinsed and weighed. The aorta was cannulated and the coronary arteries were cleared by perfusion with cold Krebs-Henseleit Buffer. The hearts were then gravity perfused with 10% formalin at mean arterial pressure (100 mmHg). Fixed hearts were immersed overnight in 10% formalin and then stored in 70% ethanol for up to 1 week before being processed. The heart was cut on a short axis plane, starting at the apex and continuing up to the base and then short axis sections were cut into half (lateral and septal wall) and embedded in paraffin wax blocks. 5µm tissue sections from 6 different levels from each sample were slide-mounted (AML Laboratories, Florida, USA).

Heart Histology: Paraffin-embedded LV samples fixed at 4 months post-banding were stained with Masson's Trichrome (Sigma-Aldrich; St. Louis, MO) to determine percentage of fibrosis. Cytoplasm and muscle fibers stain red, while collagen (fibrosis) is stained blue. 120 slides (60 lateral wall, 60 septal wall) from 10 animals (sham=4, banded=6) and from 6 different levels of the LV were utilized to obtain representative data from different regions of the heart. The stained slides were imaged at 4x magnification (Nikon Eclipse Ni, NIS Elements software; New York, USA) and analyzed using Image J software with color threshold analysis. A total of 1103 pictures were analyzed. The percentage of fibrotic tissue was determined as the collagen positive tissue out of the total stained LV tissue. In order to examine the LV fibrosis gradient, the subendocardium and subepicardium were imaged separately. 20 slides (10 lateral wall, 10 septal wall) from 10 animals (sham=4, banded=6) were examined. A total of 267 pictures were analyzed for the LV fibrosis gradient. All images were taken at 4x magnification (Nikon Eclipse Ni, NIS Elements software; New York, USA) and analyzed using Image J software with color threshold analysis. The percentage of fibrotic tissue was determined as the collagen positive tissue out of the total stained LV tissue.

To determine myocyte cross-sectional area, LV tissue sections were stained for wheat germ agglutinin (WGA; Life Technologies; Eugene, OR) and nuclei were labeled with 4’,6- diamidino-2-phenylindole (DAPI, Millipore; Billerica, MA). Confocal micrographs of all immunostained slides were acquired using a Nikon Eclipse T1 confocal microscope (Nikon Inc.; Mellvile, NY). Myocyte cross-sectional area was measured using NIH Image J software as previously described (11). A total of 6384 myocytes were counted from 15 animals (sham=7, banded=8).

Lung Tissue Collection and Processing: Following the final in vivo measurements, the animals were deeply anesthetized via a bolus of sodium-pentobarbital while ventilation continued. Prior to removing the heart as described above, the pulmonary artery was cannulated. Once the heart was removed, the right cranial apical lobe was clamped in order to obtain tissue aliquots for assessment of wet/dry weight ratio. Then, the pulmonary vasculature was perfused with cold sterile 0.9% saline ≤ 15 mm Hg until the perfusate ran clear. The left and right lungs were prepared and dissected according to a predetermined matrix to support unbiased sampling procedures (7,8). Samples (2 - ~1 cm^3) from the right lower lobe were submerged in buffered formalin saline, washed in PBS, and stored in ethanol until prepared for standard paraffin embedding. Samples (2 - ~1 cm^3) from the left lung were snap-frozen in liquid nitrogen and stored at –80°C for subsequent analyses. Tissues used to assess the wet/dry weight ratio were dried on clean gauze, diced, weighed, then oven-dried to completion (72 hr @ 70°C), and re-weighed.

Lung Histology and Tissue Protein Content Analysis: Analyses were performed while blinded to treatment group. For histological analysis, embedded lung samples were step sectioned at 500 μm intervals and a total of five, 5 μm sections from each sample were slide-mounted, and stained with hematoxylin and eosin. Lung sections were first viewed through a transparent grid matrix at low power (100x) and then changed to higher power (400x) to randomize selection. To eliminate sampling bias, every tenth grid region was then photographed and digitized. Morphometric image analysis (Image Pro Plus®, Silver Spring, MD) was performed with customized algorithms that were free of geometric assumptions to assess the expansion index (ratio of volume of gas exchange to parenchymal space) by densitometry, to count the number of open gas exchange units per fixed field size using a modification of the radial alveolar count method, to measure the alveolar area (~1000 alveoli) and the thickness of the alveolar-capillary membrane (~100 alveoli; 4 measurement points/alveolus) (7,9,10). Measurement of the perivascular cuffs was performed by a modification of a method described by Lowe, et.al. (11). Briefly, digitized images were viewed at 100x magnification through an overlaying grid matrix. Using Image J (NIH), the outer border of all of the vessels in the digitized fixed field were traced and the external diameter the vessels was measured. The outer border of all of the vessels ≤500 um and the perimeter of the corresponding perivascular cuff surrounding each of the vessels were traced. The ratio of the area of the cuff relative to the area of the vessel were analyzed as a function of group (sham vs. banded).

Lung tissue samples for total protein analysis were thawed slightly on ice, washed twice with PBS, and homogenized rapidly in 1 mL RIPA solution (50 mM Tris HCl, 150 mM NaCl, 1% Igepal, 0.5% NaDOC, 0.1% SDS) with CompleteTM protease (i.e. serine, cysteine and metalloproteases) inhibitor cocktail (Roche Diagnostics, Mannheim, Germany), prior to centrifugation at 12,000g for 10 minutes at 4°C to obtain supernatant which was measured using the method described by Bradford (12).

Real-time polymerase chain reaction (PCR): Hearts were harvested from sham (n=6) and banded (n=9) cats 4 months after surgery. Total RNA was extracted from snap frozen LV myocardial tissue utilizing TRIzol Reagent (Ambion, Thermo Scientific, Waltham, MA) and digested with DNase I (Invitrogen, Thermo Scientific, Waltham, MA) to eliminate genomic DNA. Reverse transcription (RT) reaction was performed using the SuperScript III first strand synthesis system for RT-PCR (Invitrogen, Thermo Scientific, Waltham, MA) and oligo-dt primers according to the manufacturer’s instructions. Real-time PCR was performed using the Quantifast Sybrgreen PCR kit (Qiagen, Germantown, MD) and the StepOnePlus Real-Time PCR system (Applied Biosystems, Foster City, CA). Data generated were normalized to beta-2 microglobulin (ß2M). The following primer sets were used (forward, reverse): ß2M:

5’-TTTGTGGTCTTGGTCCTGCT, 5’-TGGGTGACGGGAGTAAACCT; ACTA1: 5'-CGTGTTCCCTTCCATCGT, 5'-CGGAGCTCGTTGTAGAAGGT.

NT-proBNP: To detect the presence of serum markers of heart failure, arterial blood was collected by cephalic vein puncture at BL and 4 months post-banding. Blood was stored in spray-coated K2EDTA vacutainers, microcentrifuged at 1300 rpm for 10min, and plasma samples were sent to Idexx Laboratories (Memphis, TN) for NT-proBNP analysis.

Supplementary Figure 1: Study Protocol

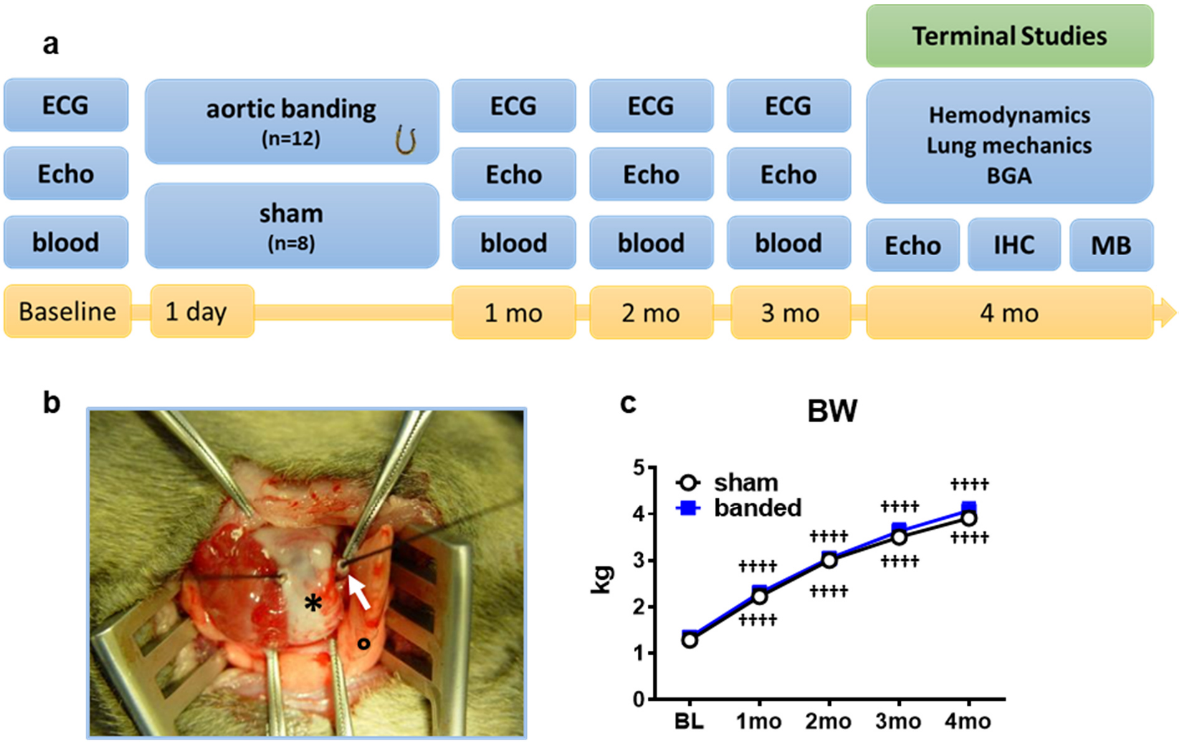

Supplementary Figure 1: Study Protocol. At baseline, electrocardiogram (ECG) and echocardiography (ECHO) were performed and blood samples were collected followed by the aortic banding or sham procedure. At 4 months post-banding or sham procedure, invasive hemodynamics and lung mechanics were recorded and tissue was harvested for molecular and histological analyses (**a**). Mini thoracotomy with lungs (°) and the pre-shaped band (white arrow) placed around the aorta (*) (**b**). Body weight (BW) did not differ between groups at any time point. † † † † <0.0001 vs BL(**c**). BGA=blood gas analysis, MB=molecular biology, IHC=immunohistochemistry.

Supplementary Figure 2: Hemodynamics

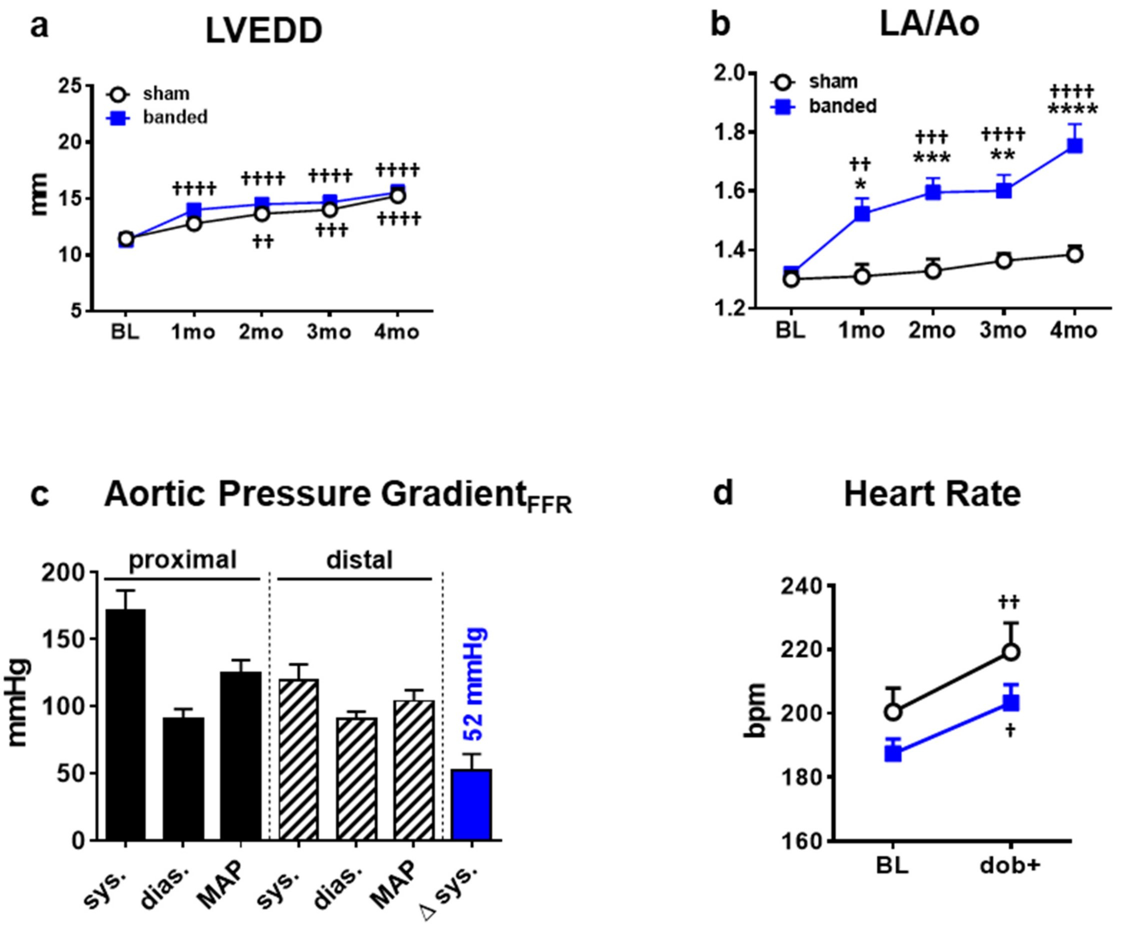

Supplementary Figure 2: Hemodynamics. The left ventricular end-diastolic diameter (LVEDD) (**a**) was comparable between sham and banded cats, while the left atrial aortic root ratio (LA/Ao) (**b**) was increased after aortic banding. Aortic pressure gradients measured with a fractional flow reserve module (FFR). Sys.=systolic pressure, dias.=diastolic pressure, MAP=mean arterial pressure, Δ sys.=systolic pressure gradient across the band, proximal and distal indicates the pressure in context to the band location (**c**). Heart rates at baseline (BL) and after dobutamine (dob+) infusion **(d)**. *p<0.05, **p<0.01, ***p<0.001, ****p<0.0001 between groups. †p<0.05, ††p<0.01, †††p<0.001, ††††p<0.0001 compared to BL.

Supplementary Figure 3: Cardiopulmonary Correlations and Lung Parameters

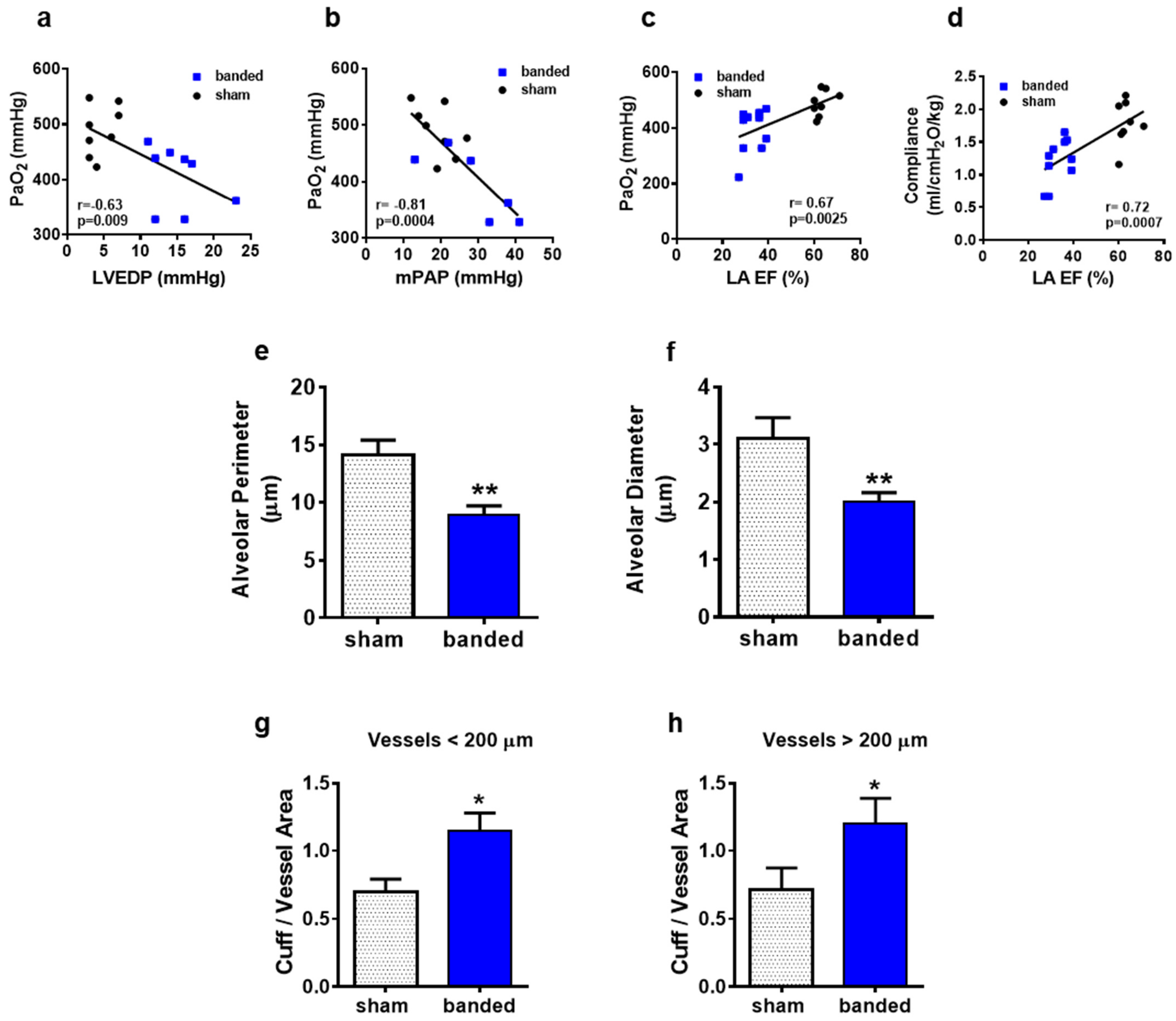

Supplementary Figure 3: Cardiopulmonary Correlations and Lung Parameters. Left ventricular end-diastolic pressure (LVEDP) and mean arterial pulmonary pressure (mPAP) inversely correlates with partial arterial oxygen pressure (PaO_2) and left atrial ejection fraction (LA EF) positively correlates with PaO_2 and respiratory compliance (**a-d**). Alveolar dimensions (**e-f**) and cuff to vessel area ratio in smaller (range: 80 – 160 µm) (**g**) and larger (range: 200 – 500µm) (**h**) vessels. *p<0.05, **p<0.01 between groups.

Supplementary References

1. Riesen SC, Schober KE, Smith DN, Otoni CC, Li X, Bonagura JD. Effects of ivabradine on heart rate and left ventricular function in healthy cats and cats with hypertrophic cardiomyopathy. Am J Vet Res 2012;73:202-12.
2. Hansson K, Haggstrom J, Kvart C, Lord P. Left atrial to aortic root indices using two-dimensional and M-mode echocardiography in cavalier King Charles spaniels with and without left atrial enlargement. Vet Radiol Ultrasound 2002;43:568-75.
3. Russo C, Hahn RT, Jin Z, Homma S, Sacco RL, Di Tullio MR. Comparison of echocardiographic single-plane versus biplane method in the assessment of left atrial volume and validation by real time three-dimensional echocardiography. Journal of the American Society of Echocardiography : official publication of the American Society of Echocardiography 2010;23:954-60.
4. Mattioli AV, Sansoni S, Lucchi GR, Mattioli G. Serial evaluation of left atrial dimension after cardioversion for atrial fibrillation and relation to atrial function. The American journal of cardiology 2000;85:832-6.
5. Linney CJ, Dukes-McEwan J, Stephenson HM, Lopez-Alvarez J, Fonfara S. Left atrial size, atrial function and left ventricular diastolic function in cats with hypertrophic cardiomyopathy. J Small Anim Pract 2014;55:198-206.
6. Koffas H, Dukes-McEwan J, Corcoran BM et al. Pulsed tissue Doppler imaging in normal cats and cats with hypertrophic cardiomyopathy. J Vet Intern Med 2006;20:65-77.
7. Wolfson MR, Hirschl RB, Jackson JC et al. Multicenter comparative study of conventional mechanical gas ventilation to tidal liquid ventilation in oleic acid injured sheep. ASAIO J 2008;54:256-69.
8. Hsia CC, Hyde DM, Ochs M, Weibel ER, Structure AEJTFoQAoL. An official research policy statement of the American Thoracic Society/European Respiratory Society: standards for quantitative assessment of lung structure. Am J Respir Crit Care Med 2010;181:394-418.
9. Wolfson MR, Greenspan JS, Deoras KS, Rubenstein SD, Shaffer TH. Comparison of gas and liquid ventilation: clinical, physiological, and histological correlates. J Appl Physiol (1985) 1992;72:1024-31.
10. Deoras KS, Wolfson MR, Searls RL, Hilfer SR, Sheffield JB, Shaffer TH. Use of a touch sensitive screen and computer assisted image analysis for quantitation of developmental changes in pulmonary structure. Pediatr Pulmonol 1990;9:109-18.
11. Lowe K, Alvarez DF, King JA, Stevens T. Perivascular fluid cuffs decrease lung compliance by increasing tissue resistance. Critical care medicine 2010;38:1458-66.
12. Bradford MM. A rapid and sensitive method for the quantitation of microgram quantities of protein utilizing the principle of protein-dye binding. Anal Biochem 1976;72:248-54.
